# Patterns of Change in Employment Status and Their Association with Self-Rated Health, Perceived Daily Stress, and Sleep among Young Adults in South Korea

**DOI:** 10.3390/ijerph16224491

**Published:** 2019-11-14

**Authors:** Eun-Sun Lee, Subin Park

**Affiliations:** Department of Research Planning, Mental Health Research Institute, National Center for Mental Health, Seoul 04933, Korea; chachagrace918@gmail.com

**Keywords:** employment status change, trajectory, latent class growth modeling, self-rated health, perceived stress, sleep

## Abstract

We identified distinct trajectories of temporal changes in employment status and investigated their association with self-rated health, perceived stress, and sleep. Data pertaining to 1228 respondents (age: 17–31 years) were extracted from the Korea Youth Panel (YP2007) survey (3rd–9th wave) datasets. Participants were either paid employees (permanent or precarious) or currently unemployed but seeking a job at baseline. Latent class growth analyses were employed to extract different classes based on the annual change in employment status (permanent/precarious/unemployed). Logistic regression analyses were performed using extracted classes as predictor variables and health-related variables at the final time-point as outcome variables. Five trajectories of employment status change were identified: stability sustained; gradually deteriorated; swiftly alleviated; gradually alleviated; instability sustained. Compared with the stability sustained group, the gradually deteriorated and gradually alleviated groups showed higher odds of perceived stress. The gradually deteriorated, instability sustained, and gradually alleviated groups showed significantly higher odds of shorter sleep than the stabilized group. We highlight the adverse health effects of prolonged unstable employment and the need for interventions to mitigate these effects.

## 1. Introduction

The last three decades have witnessed substantial socioeconomic changes in the modern societies. The phenomena of globalization, deindustrialization, and technological advances have resulted in significant transformation in the labor market, marked by increased workforce flexibility. This flexibility has further led to changes in employment conditions, thereby resulting in alternative forms of employment as opposed to the standard employment relationships. The various terms used to describe these alternative forms of employment include temporary, flexible, nonstandard, irregular, or precarious employment [1]. The term “precarious employment” refers to employment with unfavorable contracts with respect to wage, working terms and conditions, and employment insecurity [2].

Despite the lack of an internationally shared definition of precarious employment, it is a major societal concern worldwide. The estimated prevalence rates of temporary employment in OECD countries range between 1.1% and 28.8% (mean rate in 2018: 11.7%) [3]. Considering the associated poor job security, low remuneration, limited entitlements and protections, and low socioeconomic benefits involved in its nature [4], precarious employment can have a detrimental effect on health. Epidemiological studies on precarious and flexible employment have highlighted its adverse effects on both physical (e.g., increased episodes of illness, longstanding illness, increased cardiovascular risk, worsening of general health, and morbidity) and mental health (e.g., increased prevalence of depression, new-onset severe depression, anxiety, burnout, and suicidal ideation and suicide attempts) [5,6,7,8].

Of note, young adults are particularly susceptible to insecure and disadvantaged employment. The estimated global prevalence rates of temporary employment among population aged 15–24 years range from 4.51% to 71.20%; the mean prevalence rate of 25.67% is over two times the rate found among overall paid employees in 2018 [3]. This is likely attributable to their lack of work experience and fewer work opportunities in the labor market [9]. High rates of youth unemployment can also increase precarious unemployment by reducing the bargaining power of workers and their ability to decline unstable employment or poor working conditions. High rate of youth unemployment is a serious socioeconomic issue in South Korea, which may make youths more vulnerable to precarious employment. According to a study, approximately 35% young adults in South Korea (even those with doctoral degrees) start their career precariously [10]. Considering the pervasiveness of unemployment and nonstandard jobs for the youth population, the psychosocial and health problems associated with those conditions have become an important sociopolitical and scientific issue.

In this context, an increasing number of studies have examined the long-term health effects of such conditions. For instance, workers with full-time employment were more likely to show slower decline in perceived health and physical functioning [11], whereas workers exposed to chronic job insecurity showed higher odds of poorer self-rated health [12]. Similarly, when compared with individuals who remained permanently employed, those who transitioned from permanent to precarious employment or unemployment, as well as those who continued to be unemployed or precariously employed, showed a significantly higher risk of developing depression [7]. A transition from permanent to either full-time or part-time precarious employment was also associated with increased suicidal ideation among workers with no previous history of suicidal ideation [13]. Furthermore, researchers have attempted to figure out potential mechanisms underlying the link between unemployment/precarious employment and health: the life-course perspective, which emphasizes the vulnerability of, and thus the need for increased incentives for, those in the transition process to adulthood, the lack of social and economic benefits provided by standard employment, and the increased burden on males as breadwinners because of the changing social roles in modern societies [14].

Although these results have shed light on the temporal association between unfavorable employment status and health status of youth, a majority of these studies enrolled adult subjects in all age groups; therefore, the results may not be entirely applicable to young adults. Furthermore, most longitudinal studies were based on short-term data; in addition, these simply classified respondents into arbitrarily determined classes, thereby failing to identify meaningful long-term patterns that reflect actual changes in employment status and their proportions among youth. Therefore, in the present study, we sought to examine the temporal changes in employment status of young adults in South Korea using youth panel follow-up survey data.

Employment insecurity is also known to increase the risk of insomnia or general sleep disturbances, with each unit increase in job insecurity augmenting the odds of sleep difficulties by 47% [15]. In addition, stress and inadequate sleep are also valid indicators of general health, health-related complaints, and mental distress (e.g., depression and anxiety) [16,17], accounting for 39%–56% of health [18]. Based on these previous findings, we examined self-rated health, perceived daily stress, and sleep among young adult workers and explored the association of distinct trajectories of change in employment status with these variables.

## 2. Materials and Methods

### 2.1. Data Description

This study utilized data from the Korea Youth Panel (YP2007) survey. The YP2007 is a longitudinal survey conducted by the Korea Employment Information Service, which is funded by the Ministry of Employment and Labor. The YP2007 uses a representative sample of Korean youth population aged 15–29 years at baseline and collects data about the school life (e.g., type of high school and college attended, major, suspension of graduation), socioeconomic activities (e.g., job-seeking activities, past/current occupations), and family background (e.g., employment status of parents, family income) of the respondents. Accumulation of data from annual follow-ups of the initial sample provides information on temporal trajectories such as school-to-work transition and career path of youth. This information is intended to inform governmental policies for effective education, skill development, and improved social infrastructure to resolve the high youth unemployment rate and to facilitate stable employment among the youth in South Korea.

The YP2007 utilizes a computer-assisted personal interview wherein trained researchers conduct face-to-face interviews of participants using a laptop computer. A total of 10,206 participants were enrolled at the initial survey conducted in 2007; these participants are followed up annually. The latest round of survey (wave 13) is currently in progress. In addition to the regularly surveyed items, special issues such as opinions pertaining to governmental policies for youth unemployment (wave 1), disabilities or physical constraints (wave 2), or interaction with parents (wave 2) are also conducted in individual waves on an irregular basis. Questions pertaining to health- and stress-related issues have been incorporated in the survey on a triennial basis since the year 2009 (wave 3). These are intended to facilitate assessment of perceived health, health-risk behaviors, and stress.

### 2.2. Participants

Data were downloaded from the Employment Survey website. In this study, we only used data pertaining to years 2009 (wave 3, wherein health and stress were examined for the first time) to 2015 (wave 9, which is the most recent survey) in order to explore the association of change in employment status with health-related factors.

**Figure 1:**
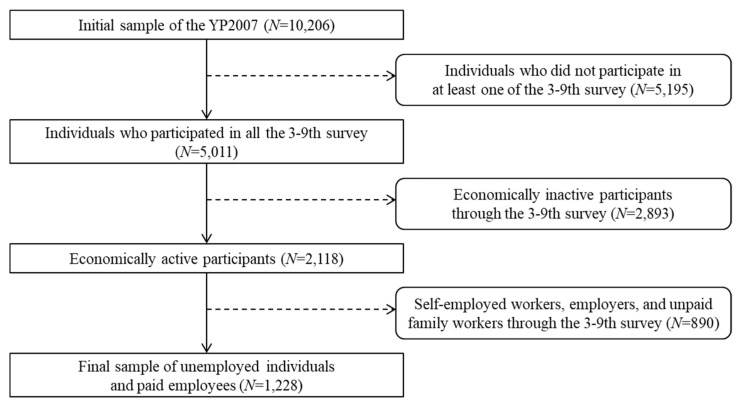
Schematic illustration of the selection criteria for the study population using the third–ninth waves of the YP2007 survey.

A schematic illustration of the selection criteria for the present study is presented in Figure 1. First, individuals who responded to all the surveys from wave 3 (2009) to wave 9 (2015) were screened (*N* = 5011). Second, among these individuals, those who reported being unemployed as well as expressed a lack of intention to find work (i.e., economically inactive) in at least one of the seven waves were excluded (*N* = 2118). Economically active people comprise unemployed individuals seeking work and employed individuals (paid or unpaid). Since the employment status of employees was the focus of our study, we included only paid employees among the employed, and excluded self-employed workers, employers, and unpaid family workers at all time-points, for whom the quality of employment could not be estimated. As a result, the final sample of the present study consisted of 1228 individuals who were unemployed or paid employees.

This study was reviewed and approved by the Institutional Review Board of the National Center for Mental Health (No. 116271-2019-35).

### 2.3. Measurements

Of the paid employees, those under the following work types were classified as “precarious” employees: (1) fixed-term workers; (2) temporary workers; (3) workers in special types of employment; (4) home-based workers; (5) dispatched workers; (6) subcontract workers; (7) on-call workers; and (8) part-time workers. Other paid workers who did not come under the above categories were classified as “regular” workers (i.e., full-time permanent workers), whose contract periods are not pre-determined and who are guaranteed to continue to be employed unless there are special circumstances. As a result, employment status was coded as a three-category variable: 1 = permanent employment; 2 = precarious employment; and 3 = unemployment.

Self-rated health, perceived daily stress, and hours of sleep at the 9th investigation were adopted as outcome variables. Self-rated health was assessed using the question “How do you think about your current health status?” The responses were recorded using a 5-point Likert scale (1 = *very good*; 5 = *very poor*). The perceived daily stress was measured with the question “How much stress do you experience in your daily life?” The responses were recorded using a 4-point Likert scale (1 = *very much*; 4 = *little*). With regard to hours of sleep, participants were required to directly answer how much time they sleep in a day on average.

### 2.4. Statistical Analyses

**Figure 2:**
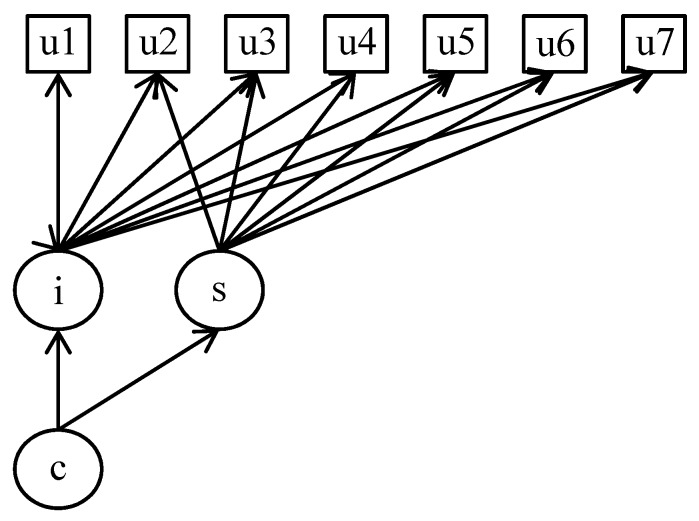
Model for latent class growth analysis. Note. u1–u7 indicate three-category outcome (i.e., employment status).

We used latent class growth analysis (LCGA) models to identify distinct groups based on homogeneous trajectories of employment status over seven years. LCGA is a group-based semiparametric approach that classifies individuals into homogenous groups or classes based on the similar pattern of temporal changes with respect to a variable of interest [19,20]. By specifying and comparing consecutive LCGA models in which the number of latent classes is increased successively, we can identify the model that shows the best-fit with the data. Figure 2 presents the LCGA model in which latent classes are explored using tri-categorical variables across seven time-points (u1–u7). In the model, i (intercept) and s (slope) are latent growth factors, which are formed by the observed variables u1–u7, and then, the latent classes c are determined by i and s.

To determine the optimal number of latent trajectory clusters, we utilized multiple criteria including Bayesian information criterion (BIC), sample-size adjusted Bayesian information criterion (SSABIC), entropy, bootstrap likelihood ratio test (BLRT), and proportions for the latent classes in combination with interpretability of the extracted classes. First, the information indices BIC and SSABIC are goodness-of-fit measures, lower scores of which indicate better model fit. Second, entropy is a standardized measure of the quality of classification; its value ranges from 0 to 1 (1 indicates perfect classification of participants). Third, BLRT provides statistical significance value for comparison between the model with *k* classes and the other model with *k-1* classes. BLRT *p*-value of less than 0.05 suggests that the model with *k* classes exhibits a significantly improved model fit as compared with the model with one class fewer, and thus should be accepted. Finally, we considered the proportions of individuals in the latent classes against the entire sample. A model is considered to be acceptable when each latent class contains at least 5% of the total sample [21]. To identify statistically unbiased and interpretable classes, we only considered models wherein the proportion in each class was at least 5%. The LCGA was conducted using Mplus version 6.0 (Muthén, & Muthén, Los Angeles, CA, USA).

We then performed bivariate logistic regression analyses with the latent classes extracted from the LCGAs as the predictor variable and the self-rated health, perceived daily stress, and hours of sleep reported in wave 9 as the main outcome variables, after adjusting for age, sex, education level, income paid per unit time, and marital status at wave 9. Of all the variables, missing values were found only for pay per unit time (*N* = 24, 2%); for this we used the computed values estimated from Expectation Maximization. Among the outcome variables, those that were originally measured on a four- or five-point Likert scale were dichotomized into two categories to perform bivariate logistic regression analyses. Self-rated health was coded as 0 if an individual answered *fair*, *good*, or *very good*, and 1 if he/she reported *poor* or *very poor* general health. Perceived daily stress was coded as 0 if the response was *little* or *a little*, and 1 if the response was *pretty much* or *very much*. The response to average hours of daily sleep was grouped into two categories based on the median value of 7 h: 0 indicates daily sleep *≥7 h* and 1 indicates daily sleep *<7 h*. Descriptive statistics and multiple bivariate logistic regression analyses were conducted using SPSS version 21.0 (IBM Corp, Armonk, NY, USA).

## 3. Results

A total of 1228 respondents (522 females and 706 males) were included in the analyses. The mean age of respondents in wave 3 was 26.94 ± 2.79 years (range, 17–31). The characteristics of respondents are summarized in Table 1.

To identify the optimal number of different trajectory classes, we compared fit indices for the latent class growth models with 2–6 latent classes. Fit indices for each model are presented in Table 2. Goodness-of-fit indices such as BIC and SSABIC improved from the 2-class through the 5-class models but worsened at the 6-class model. Entropy value was highest in the 2-class model, decreased gradually through the 3- and 4-class models, and then showed a rebound in 5- and 6-class models. BLRT values indicated gradually improved model fit over 2- to 5-class models, whereas the 6-class model no longer yielded incremental model fit. Participants allocated to each latent class in all models, except for the 6-class model, accounted for no less than 5%. We chose the 5-class model as the most likely representation of trajectory patterns; in this model, the BIC and SSABIC were the lowest, BLRT was significant, and all the percentage of subjects in each class exceeded 5%. The 5-class model showed relatively high accuracy of classification with mean latent class posterior probabilities ranging between 0.725 and 0.924.

**Figure 3:**
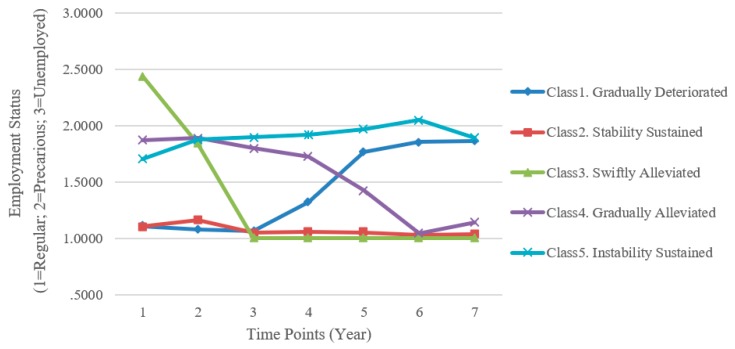
Five course trajectories identified by latent class growth analysis.

For descriptive purpose, we labeled the latent classes based on the pattern of changes in employment status (Figure 3). Class 1 was named “gradually deteriorated” group (*N* = 94, 7.65%) as individuals in this group were initially regularly employed but gradually transitioned to unfavorable employment conditions over a period of seven years. Class 2 was “stability sustained” group (*N* = 808, 65.80%), in which the employment status remained steady on average over seven years. In contrast, class 3, “swiftly alleviated” group (*N* = 85, 6.92%), showed a sudden rise in employment conditions at the third time-point after being initially unemployed or employed under precarious jobs for two years. Participants in class 4 (*N* = 114, 9.28%) showed steady improvement in employment conditions and finally achieved standard employment at the sixth time-point; therefore, this class was labeled as “gradually alleviated” group. Class 5 (*N* = 127, 10.34%) was termed “instability sustained” group; in this group, the employment status remained precarious on average over all time-points.

Table 3 presents means and standard deviations of health-related variables at the final time-point for extracted classes. Table 4 shows the results of bivariate logistic regression analyses. After adjusting for age, sex, education level, income per unit time, and marital status, the self-rated health in the stability sustained group was not significantly different than that in the other four classes. However, gradually deteriorated group showed significantly higher odds of experiencing greater stress (adjusted odds ratio [AOR] = 2.06, 95% confidence interval [CI] = 1.23–3.46), while the gradually alleviated group showed a tendency for greater stress (AOR = 1.99, 95% CI = 0.99–3.99, *p* = 0.052). With regard to the lack of sleep (<7 h daily), gradually deteriorated group (AOR = 1.60, 95% CI = 1.03–2.48), gradually alleviated group (AOR = 2.19, 95% CI = 1.20–3.99), and instability sustained group (AOR = 1.80, 95% CI = 1.03–3.16) had greater odds of reporting less hours of daily sleep than the other groups did.

## 4. Discussion

The present study identified distinct temporal trajectories of employment status among young adults in South Korea and examined their association with health-related indicators using 7 year follow-up data derived from the YP2007. The findings and implications of this study are discussed below.

Latent class growth analyses identified five distinct classes depending on the temporal change in employment status: stability sustained, gradually deteriorated, swiftly alleviated, gradually alleviated, and instability sustained. This result is notable in at least two points. Firstly, despite over half of respondents maintaining permanent employment, over 10% of economically active respondents remained precariously employed across all time-points. Approximately 10% of young adults who had been precariously employed at baseline were unable to fall out of such unfavorable conditions; this likely indicates the rigid socioeconomic structure of South Korea which imposes a barrier to career advancement of young adults. Although young adulthood is a period of constant transition in working positions [22], changes in the labor market that may not allow an individual to exercise his discretion and explore different career paths and employment status may result in frustration and hopelessness by limiting the possibility to explore and realize their potential. In this context, the number of young adults affected by health inequalities in South Korea may be larger than expected. This calls for appropriate policy-level and health interventions.

Secondly, previous studies have not been able to differentiate between steady and sudden improvements in employment status using conventional methods due to the relatively short follow-up period. In this study, we identified two groups as distinct classes with statistically meaningful differences in trajectories; our results suggest that the two groups may have disparities in other important aspects such as physical health and mental health. Although both groups experienced improvements in employment conditions, differences in the length of periods during which they were exposed to unfavorable working conditions (2 and 5 years for sudden and steady improvement group, respectively) may have contributed to the heterogeneity. This is in keeping with a previous study, in which individuals with longer periods of exposure to temporary employment showed a tendency for increased risk of suboptimal self-rated health and sleep quality [23]. This finding highlights the importance of considering the adverse effect of a long period of precarious employment even in individuals who finally achieved regular employment.

With regard to the association between the classes and the health-related indicators, individuals with gradual deterioration or alleviation in working conditions were at two-fold higher risk of heightened daily stress. Considering that nonregular forms of employment impose additional burden on workers with respect to tasks (e.g., time pressure) and employment (e.g., effort to sustain employment) [24], transition to disadvantaged employment conditions may have led to higher stress and strain. Increased stress in the gradually alleviated group is noteworthy in that this may imply lingering negative effects of prolonged precarious employment on the mental well-being even after improvement in employment status.

On the other hand, the instability sustained group (characterized by sustained unfavorable employment status) did not significantly differ from the stabilized group in terms of perceived daily stress. Considering that perception of stress is a reaction to external stressors, individuals in the instability sustained group (exposed to extended periods of precarious employment, more frequent loss of jobs, and economic strain) may have been become too accustomed to chronic stressors to explicitly notice and report the perceived stress. This is in line with the apathy observed in individuals who are chronically exposed to a stressful environment [25]. Given this finding, psychological distress derived from chronic exposure to precarious employment may be under-recognized and manifest predominantly as alternative forms of distress, such as psychosomatic symptoms [26].

In addition, all groups, except for those who showed swift alleviation, had significantly higher odds of inadequate sleep compared with the stabilized group. Relatively shorter hours of sleep in all groups exposed to prolonged precarious employment may be attributable to the negative subjective experiences associated with employment insecurity; these include concerns pertaining to job stability, prospective job opportunities, or financial problems. The consequent emotional and physiological arousal may prevent initiation and/or maintenance of sleep [15]. This result is also significant in that less than 7 h of sleep per night is associated with increased risk of negative health outcomes, including hypertension, weight gain and obesity, diabetes, death [27], and depression [28]. This finding is consistent with that of a previous study, in which odds of experiencing depressive symptoms associated with perceived job insecurity peaked after approximately 3–4 years of prolonged exposure to unstable employment [29]. Given these results, preventative intervention and proper guidance targeting for stress and sleep management among young adults in prolonged precarious employment need to be introduced and provided at government level. In addition, preexisting mental health services of institutions and companies should be expanded to include precarious employees, who were found to be more vulnerable but underserved.

The extracted classes showed no significant differences with respect to self-rated general health; this is inconsistent with a previous study that found a positive association between prolonged precarious employment and poorer self-rated health [30]. The single item used in the YP2007 is commonly adopted for assessment of self-rated health in population surveys and empirical research; however, its ambiguous and subjective nature renders it prone to person-specific heterogeneity and bias depending on different populations or cultures [31]. This may have led to the inconsistent findings. In addition, given that adverse effects of precarious employment on health can vary depending on the work conditions (e.g., full-time vs. part-time working under precarious contracts) [32] or type of occupation (e.g., care service workers) [33], more specific studies that take into account such variables are needed to elucidate the association.

Some limitations of our study should be considered while interpreting the findings. First, as we utilized data from respondents who participated in every wave, a sample selection bias may have affected the results. Furthermore, we excluded all those respondents who were economically inactive at least once during the 7 year period as their employment status could not be defined at such time-points. Considering that precariously employed or unemployed people are more likely to become economically inactive over time when compared with the regularly employed, uniform exclusion of economically inactive people may have biased our study participants toward those in more favorable employment status. In addition, given that approximately 28% of the economically inactive population were involuntarily dormant due to external conditions, such as expiration of preexisting contracts, sudden lay-off, shutdown of their workplaces, or a lack of desired positions [34], the number of unemployed people may have been underestimated in the present study.

Second, the negative impact of precarious employment can vary depending on whether an individual’s choice to work contingently was voluntary. For instance, maternal nonparticipation and part-time employment due to family constraints (e.g., childcare) had a significantly negative impact on individuals’ life satisfaction [35]. In contrast, individuals who feel that they voluntarily chose precarious employment were found more likely to experience better working conditions, such as higher income or being in clerical, professional, or managerial positions, and to have more positive organizational experiences than those who feel that they are forced to work precariously due to a lack of permanent jobs [36,37]. Given that 53% of precariously employed workers opted for precarious jobs in South Korea [38], failing to consider the extent of voluntariness may have precluded precise inferences about the impact of prolonged precarious employment on health-related variables.

Lastly, the current study could not identify the pathways that lead to differences in outcome variables among the different groups. For instance, one qualitative study revealed that the lack of coping resources (e.g., control, support, trust, and equity) has a significant influence on the effect of precarious employment on the mental well-being of employees [39]. Similarly, psychosocial aspects of employment, such as perceived job insecurity, psychological demand, lack of entitlement, and low availability of alternative jobs, were shown to putatively mediate the effect of precarious employment on ill health [40,41]. In order to develop interventions to mitigate the negative impact of precarious employment, further studies should seek to identify the objective and subjective factors (and their possible mechanisms) involved in incurring or maintaining the adverse effects.

## 5. Conclusions

To the best of our knowledge, this is the first study that identified statistically meaningful patterns of temporal change in employment status over a relatively long period of time and their association with health and health-related variables in young adults in South Korea. Our results indicated that approximately 27% of young workers have been exposed to disadvantaged employment conditions for over 4 years at some point across the seven-year study reference period; these were more likely to experience higher stress and a lack of sleep. Our findings highlight the need to develop socioeconomic policies to detect and protect young workers who are vulnerable to prolonged disadvantaged positions. In addition, there is a need to develop preventative psychosocial interventions targeting young individuals who remain precariously employed for a long period of time and thus are at higher risk of negative health outcomes.

## Figures and Tables

**Table 1:** Characteristics of the study population at wave 9 of YP2007 survey.

Characteristics	*N* (%)
Education level	
High school and below	252 (20.6)
College	400 (32.6)
University	530 (43.2)
Graduate school and above	46 (3.7)
Marital status	
Married	618 (50.3)
Single	606 (49.3)
Divorced	4 (0.3)
Average income paid per unit time	
<10,000 won	97 (8.1)
10,000 won–less than 20,000 won	816 (67.8)
20,000 won–less than 30,000 won	247 (20.5)
≥30,000 won	44 (3.7)
Refused to answer	24 (2.0)

**Table 2:** Model fit statistics for determining the number of latent classes in the context of change in employment status.

Models	Log Likelihood	BIC	SSABIC	Entropy	BLRT	Proportions for the Latent Classes (%)					
						1	2	3	4	5	6
2-Class	−4436.763	8916.206	8897.147	0.846	0.000	25.65	74.35				
3-Class	−4370.017	8804.053	8775.465	0.840	0.000	18.08	9.68	72.25			
4-Class	−4296.295	8677.947	8639.830	0.779	0.000	12.75	62.16	14.41	10.68		
5-Class	−4259.782	8626.261	8578.614	0.806	0.000	9.64	62.62	6.46	10.71	10.58	
6-Class	−4292.738	8713.512	8656.337	0.826	0.100	12.38	10.64	0.00	14.93	0.27	61.79

Note. BIC, Bayesian information criterion; SSABIC, sample-size adjusted Bayesian information criterion (n* = (n + 2)/24); BLRT, bootstrap likelihood ratio test.

**Table 3:** Mean and standard deviation of health-related variables at final time-point.

	Gradually Deteriorated	Stability Sustained	Swiftly Alleviated	Gradually Alleviated	Instability Sustained
Self-rated health	2.00 (0.57)	1.97 (0.62)	1.91 (0.67)	2.04 (0.59)	1.94 (0.47)
Perceived daily stress	2.90 (0.51)	2.68 (0.60)	2.78 (0.59)	2.88 (0.48)	2.80 (0.52)
Hours of sleep	6.80 (0.75)	6.93 (0.77)	6.90 (0.75)	6.80 (0.82)	6.80 (0.92)

**Table 4:** Association between change in employment status and poor perceived health, perceived daily stress, and health-related behaviors.

Variables	Gradually Deteriorated		Swiftly Alleviated		Gradually Alleviated		Instability Sustained	
	AOR	*p*	AOR	*p*	AOR	*p*	AOR	*p*
	(95% CI)		(95% CI)		(95% CI)		(95% CI)	
Self-rated poor health	1.03	0.973	0.58	0.664	1.36	0.762	0.52	0.599
	(0.23–4.59)		(0.05–6.63)		(0.19–9.92)		(0.05–5.87)	
Perceived daily stress	2.06	0.006	1.13	0.751	1.99	0.052	1.70	0.117
	(1.23–3.46)		(0.54–2.36)		(0.99–3.99)		(0.88–3.28)	
Lack of sleep (<7 h daily)	1.60	0.036	1.52	0.171	2.19	0.010	1.80	0.041
	(1.03–2.48)		(0.84–2.76)		(1.20–3.99)		(1.03–3.16)	

Abbreviations. AOR, odds ratio adjusted for age, sex, education level, income per unit time, and marital status; 95% CI, 95% confidence interval. Note. In logistic regression analyses, stability sustained group was treated as the reference.
